# Assessing gastric cancer risk using the OLGA and OLGIM systems in Republic of Moldova

**DOI:** 10.3389/fmed.2025.1563889

**Published:** 2025-03-18

**Authors:** Adriana Botezatu, Radu-Alexandru Farcas, Simona Grad, Dan-Lucian Dumitrașcu, Nicolae Bodrug, Massimo Rugge

**Affiliations:** ^1^Nicolae Testemiţanu State University of Medicine and Pharmacy, Chișinău, Moldova; ^2^Second Department of Internal Medicine, University of Medicine and Pharmacy Iuliu Hatieganu, Cluj-Napoca, Romania; ^3^Department of Medicine DIMED, University Hospital of Padua, Padua, Italy

**Keywords:** chronic atrophic gastritis, intestinal metaplasia, OLGA/OLGIM, gastric cancer, gastric dysplasia

## Abstract

**Background:**

Gastric cancer is still an important public health problem. Efforts have been made to lower its prevalence globally. The Operative Link on Gastritis Assessment (OLGA) and operating link for gastric intestinal metaplasia (OLGIM) histological grading systems have been proposed to identify individuals with types of gastritis that have the potential to become malignant.

**Aim of the study:**

Our study was conducted to assess the value of OLGA and OLGIM systems in the diagnosis of gastric precancerous lesions, in the Moldovan population.

**Methods:**

In a prospective study, 142 consecutive patients with chronic atrophic gastritis (CAG) from a larger group of patients referred to upper gastrointestinal endoscopy for dyspeptic symptoms or gastric cancer screening was investigated. The sample was divided into three groups: (a) CAG without intestinal metaplasia and gastric dysplasia; (b) CAG with intestinal metaplasia; (c) CAG with gastric dysplasia according to the morphological type of the lesion. GastroPanel biomarkers were correlated with OLGA and OLGIM stages.

**Results:**

There was a direct, moderate and statistically significant correlation between types of CAG and OLGA stages (*p* < 0.001), a direct, weak and statistically significant correlation between forms of chronic atrophic gastritis and OLGIM stages (*p* < 0.001). A statistically significant reduction in Pepsinogen I and the Pepsinogen-I/Pepsinogen-II ratio was observed alongside an increase in the stages of the OLGA and OLGIM systems.

**Conclusion:**

OLGA and OLGIM systems are useful tools in diagnosing CAG. This is the first study assessing the use of this systems in the Moldovan population.

## Introduction

Chronic atrophic gastritis (CAG) is the elective stomach cancerization field; in gastric atrophic microenvironment, gastric dysplasia (GD) is the major precursor lesion of gastric cancer (GC). The grade (i.e., histological score) and the topography (antral *versus* oxyntic) of atrophic lesions consistently correlate with the GC risk. The combined endoscopy/histological assessment of these lesions may be confidently applied in clinical strategies for GC secondary prevention (1–3).

In 2005, an international group of gastroenterologists and pathologists developed and proposed a staging system for atrophic gastritis (OLGA - Operative Link on Gastritis Assessment). This system serves as “global” histological measure of the severity/extent of gastric atrophy and consistently correlate with the single patient GC risk (4–6). Based on the same clinico-pathological rationale, the OLGIM (Operative Link on Gastric Intestinal Metaplasia Assessments) system restricts the atrophy score only to intestinal metaplasia (7, 8).

Both the OLGA and OLGIM systems apply a five-tiered staging scale (0, I, II, III, IV) and reliably associate a significantly increased GC risk to stages III and IV (high-risk stages). This risk stratification enables the identification of the patients on whom GC secondary prevention strategies should be applied. By limiting the atrophy assessment only to its IM-component, the OLGIM system potentially exhibits lower sensitivity in detecting individuals at high risk of GC (7, 8).

The GastroPanel (GP) is a non-invasive diagnostic test relying on the combined serological detection of three gastric functional biomarkers (pepsinogen I [Pg I] pepsinogen II [Pg II], gastrin-17 [G17]), along with ELISA (IgG) testing for IgG anti-*Hp* antibodies. Over the past decade, GP has emerged as a valuable non-invasive test for diagnosing both AG and (current or previous) *Helicobacter pylori* (*Hp*) infection. Recent studies have demonstrated its efficacy as both an individual diagnostic tool and as a population screening and surveillance method (9–13).

This prospective study primarily aims to assess the prognostic value of the OLGA and OLGIM systems in a cohort of endoscopy patients recruited in the Republic of Moldova. The secondary aim of this study was to correlate GastroPanel values with the OLGA and OLGIM systems.

This study marks the initial evaluation of risk assessment tools within the Moldovan population.

## Patients and methods

### Patients

Between 2018 and 2021, 1,150 patients underwent upper digestive endoscopy at the Department of Internal Medicine of the Nicolae Testemitanu State University of Medicine and Pharmacy of the Moldova Republic.

Eight hundred ninety-four patients were referred for dyspeptic symptoms, 201 for GC screening, and 55 due to a GC family history. One hundred seventy-three out of 894 patients were diagnosed with atrophic gastritis. Of those diagnosed, 142 met the inclusion criteria (described below) for this study. Following confirmation of eligibility, informed consent to be involved in the present study was obtained from all participants.

Inclusion criteria were: informed consent, previously confirmed CAG through morphological assessment, age 18 years or older, the diagnosis history less than 5 years. Exclusion criteria were: individuals under 18 years of age, those with malignant tumors, and those with severe systemic comorbidities impacting the course of the underlying disease such as central nervous system pathology, organic diseases of the endocrine glands, severe heart failure, severe hepatic dysfunction, severe renal dysfunction, severe lung dysfunction, hematological disorders, pregnant, lactating, and breastfeeding women were excluded, along with patients with severe coagulation disorders (INR > 3; platelets <30,000/mm3), active gastrointestinal bleeding, a history of gastric surgery, those who declined participation and did not provide informed consent.

### Study protocol

Patients’ data were collected through a structured questionnaire, extraction from medical records, and analysis of results from initial and follow-up visits. A complete physical examination was performed and instrumental, and laboratory investigations were also conducted. The obtained findings were comparatively analyzed across all three study groups.

Blood was collected from the patients in fasting condition, after 12 h of fasting. Blood samples were immediately centrifuged at 4° C, obtaining 5 mL of serum (within 2 h of collection) at −70 ° C until testing (within 6 months). GastroPanel biomarkers (PG-I, PG-II, G-17 and *Hp*-IgG) were determined with commercial GastroPanel® enzyme immunoassays (Biohit Oyj, Finland) at the Eurolab Laboratory and Medical Center, Chisinau, Republic of Moldova, according to the manufacturer’s instructions, and the PGI:PGII was calculated.

### Serology

The active *Hp* infection was assessed by histological evidence of Hp infection by Giemsa staining, (2) urease test and (3) HP-IgG antibodies. For the purpose of diagnosing CAG, upper gastrointestinal endoscopy and histopathological examination of the biopsy samples were performed.

### Esophagus-gastro-duodenoscopy

To exclude inter-observer variability, Gastroscopies were performed by a single endoscopist with experience in NBI technique, on the Olympus® Evis Exera III endoscopic system, using a high-performance endoscope model GIF-HQ190 (Olympus Medical SysteCorp, Tokyo, Japan). All EGDs were performed with intravenous anesthetic support and spontaneous breathing (Atropine, Dormicum, and Propofol). In all patients, biopsy specimens were obtained from 12 standardized sites: 4 from the antrum, 4 from the corpus (2 from the lesser curvature and 2 from the greater curvature), 2 from the angulus, and 2 from the cardia. Additional biopsy samples were obtained from any focal lesion. All the biopsy specimens were identified according to their topography and submitted in separated vials (3, 14).

Endoscopic atrophic changes in the gastric body were diagnosed based on the discoloration of the atrophic area with the transparency of blood vessels. Gastric atrophy was endoscopically assessed based on the topography of the Kimura-Takemoto atrophic border. Four “major” endoscopic topographical phenotypes of atrophy have been considered: (a) C0 (i.e.: no atrophy); (b) C1-C2 (i.e.: mild atrophy); (c) C3-O1 (i.e.: moderate atrophy); (d) O2-O3 (i.e.: severe atrophy) (15, 16).

### Histology

The histological assessment included: (a) lymphomonocytic and polymorphs ([PMN]; i.e.: “activity”) infiltrate in the lamina propria as well within the glandular lumen; (b) mucosal atrophy (OLGA including non-metaplastic and metaplastic components; OLGIM only including IM); (c) gastric dysplasia (low- and high-grade); (d) gastric cancer. The histology assessment grounded on internationally validated criteria (17, 18). Based on the histological score of the histological variables, all case were staged according to the current OLGA and OLGIM criteria; OLGA/OLGIM stages 0-I-II were considered low-risk GC stages; OLGA/OLGIM stages III-IV were considered as high-risk GC stages (1, 3, 14). *Helicobacter pylori* infection was histologically assessed based on hematoxylin–eosin and Giemsa (modified for *H. pylori* detection) stains.

All biopsy specimens were histologically assessed by two expert GI-pathologists.

### Statistics

Primary data processing was performed using the functions and modules of SPSS version 16.0 for Windows (SPSS Inc., Belmont, CA, United States, 2008) and Microsoft Office Excel 2019 on the personal computer through descriptive and inferential statistical procedures. To estimate the significant differences between the means of two groups, the t test for independent samples was used, and between the group means - the t test for pair-samples. For multiple comparisons (3 or more) we used the analysis of variance (One-Way ANOVA) with the application of post-hoc tests or the non-parametric Kruskal-Wallis test with the application of Bonferroni correction. Correlations were expressed by the rho Spearman coefficient. The 2 × 2 contingency chi-square *χ*^2^ test was used to compare categorical measures. A level of *p* < 0.05 was considered statistically significant.

### Ethics

The Ethics Committee at the Public Institution Nicolae Testemitanu State University of Medicine and Pharmacy, Chisinau of the Moldova Republic formally approved the study protocol (No. 38, dated June 17, 2019).

## Results

Based on the histology, the study sample included three sub-groups: *Group 1*: 51 cases featuring only non-metaplastic atrophy (i.e., no-IM); *Group 2*: 51 cases with atrophic IM-positive phenotype; *Group 3*: 40 cases with CAG and GD. At the time of enrollment, the socio-demographic characteristics (age, living environment, educational level, marital status, socio-professional category) were similar in patients from all three study groups. However, there was a tendency for age to increase as the severity of CAG progressed: the mean age in study group 1 was 54.94 ± 1.9 years, in study group 2 it was 57.39 ± 1.4 years, and in study group 3 it was 59.45 ± 1.7 years (*p* > 0.05). In all cases, an examination of the mucosa was performed under advanced imaging: HD-Near Focus-WLE-NBI +. Group 3, comprising 40 patients with CAG and GD, consisted of 1 patient with high-grade dysplasia and 39 patients with low-grade dysplasia. In the current cohort of 142 patients with CAG, *Hp* was positive in 113 (79.57%).

Analysis of the prevalence of OLGA system stages among patients in the study subgroups revealed significant differences. OLGA stage I was notably more common in study group 1 compared to study group 2 (29.4% vs. 13,7%, respectively; *p* < 0.001), and stage II OLGA was more prevalent in study group 1 (41.2% vs. 7.5%, respectively; *p* < 0.001) and study group 2 (58.8% vs. 7.5%, respectively; *p* < 0.01) compared to study group 3. Stage III OLGA was significantly more frequent in study group 3 compared to study group 1 (50.0% vs. 27.5%, respectively; *p* < 0.001) and study group 2 (50.0% vs. 27.5%, respectively; *p* < 0.01), while stage IV OLGA was more common in study group 3 compared to study group 1 (17.5% vs. 2.0%, respectively; *p* < 0.01) and study group 2 (17.5% vs. 0%, respectively; *p* < 0.01; Table 1).

**Table 1:** OLGA stages prevalence of in 141 patients with gastric atrophy.

OLGA STAGES (case number; %)	Group 1 Atrophy IM-negative		Group 2 Atrophy IM-positive		Group 3 Atrophy coexisting dysplasia		*p*-value
	*N*	%	*N*	%	*N*	%	
I (32; 22,5%)	15	29,4	7	13,7	10	25,0	1–2**
II (54; 38.0%)	21	41,2	30	58,8	3	7,5	1–3**, 2–3*
III (48; 33.8%)	14	27,5	14	27,5	20	50,0	1–3*, 2–3*
IV (8; 5.6%)	1	2,0	0	0	7	17,5	1–3**, 2–3**

Significant differences *p < 0.05; **p < 0.01; ***p < 0.001. The prevalence of OLGA stage I-II *versus* III-IV was 60.5 and 39.4%, respectively.

Analysis of the distribution of OLGIM system stages among patients in the study subgroups revealed one statistically significant difference. OLGIM stage I was more prevalent in study group 2 compared to study group 3 (51.0% vs. 10.0%, respectively; *p* < 0.001; Table 2).

**Table 2:** Prevalence of OLGIM stages in patients with IM positive gastric atrophy.

OLGIM STAGES (case number; %)	Group 2 Atrophy IM-positive		Group 3 atrophy coexisting dysplasia		*p*- value
	*N*	%	*N*	%	
0 (52; 36.6%)	–	–	1	2.5	NS
I (30; 21,1%)	26	51.0	4	10,0	2–3**
II (48; 33.9%)	21	41,2	27	67,5	NS
III (8; 6.3%)	2	3,9	7	17.5	NS
IV (3; 2.1%)	2	3.9	1	2,5	NS

Significant differences: *p* < 0.05; **p < 0.01; ***p < 0.001; NS, Not significant. The prevalence of OLGA stage I-II *versus* III-IV was 60.5 and 39.4%, respectively.

The comparative analysis of OLGA stages with OLGIM stages in the general study group found that the vast majority of cases with low risk of developing GC according to the OLGA system (97.7%) coincided with cases with low risk of developing GC according to OLGIM system (Table 3).

**Table 3:** Relationship between OLGA and OLGIM system stages in patients in the study group.

OLGA staging	OLGIM staging			
	Stages with low GC risk (stages I-II)		Stages with high CG risk (stages III-IV)	
	*N*	%	*N*	%
Stages with low GC risk (stages I-II)	84	97,7	2	2,3
Stages with high CG risk (stages III-IV)	47	83,9	9	16,1

The *χ*^2^ *Mantel–Haenszel* test indicates a strong and statistically significant link (*χ*^2^ = 25.78, *p* < 0.001) between the results of the OLGA system and the results of the OLGIM system. The downgrade of high-risk OLGA stages to lower-risk OLGIM stages has been noted in other studies. For this reason, the assessment of gastric mucosal changes should include the assessment of all the phenotypical changes included in the atrophy spectrum of mucosal atrophy, and in patients with low OLGA, IM should be considered as a high-risk marker for GC (3).

Our findings demonstrate a significant decline in PG-I (*p* < 0.001) and PGI:PGII (*p* < 0.001) values coinciding with the worsening of CAG according to OLGA and OLGIM stages. Analyzing the serological parameters across the general study group based on OLGA system stages revealed a consistent and statistically significant decrease in PG-I (from 79.48 ± 3.8 μg/L in stage I to 47.39 ± 10.1 μg/L in stage IV) and PGI:PGII (from 6.58 ± 0.7 in stage I to 2.92 ± 0.2 in stage IV; Table 4). Similarly, analysis based on OLGIM system stages showed a successive and statistically significant reduction in PG-I (from 71.65 ± 3.6 μg/L in stage I to 39.88 ± 3.3 μg/L in stage IV) and PGI:PGII (from 5.77 ± 0.4 in stage I to 2.89 ± 0.4 in stage IV; Table 4). A moderate but statistically significant positive correlation was observed between forms of CAG and OLGA stages (*ρ* = 0.48, *p* < 0.001). Additionally, a strong and statistically significant direct correlation was found between CAG forms and OLGIM stages (*ρ* = 0.89; *p* < 0.001; Tables 5, 6.

**Table 4:** Serology findings (mean values) by OLGA and OLGIM staging systems.

Serological variable	OLGA I	OLGA II	OLGA III	OLGA IV	*p*- values	OLGIM O	OLGIM I	OLGIM II	OLGIM III	OLGIM IV	*p*- values
PG-I (μg/L)	79,48 ± 3,8	68,70 ± 3,5	46,89 ± 2,3	47,39 ± 10,1	1–2** 1–3*** 1–4** 2–3*** 2–4**	71,65 ± 3,6	59,10 ± 3,1	51,66 ± 3,8	33,01 ± 2,4	39,88 ± 3,3	0–1** 0–2*** 0–3*** 1–3*** 2–3**
PG-II (μg/L)	12,77 ± 0,8	14,12 ± 0,3	14,98 ± 0,8	16,35 ± 3,8	*NS*	14,47 ± 0,9	14,90 ± 0,6	14,88 ± 0,6	14,30 ± 0,5	14,39 ± 0,4	*NS*
PGI:PGII	6,58 ± 0,7	4,98 ± 0,5	3,80 ± 0,4	2,92 ± 0,2	1–2** 1–3*** 1–4*** 2–3*** 2–4***	5,77 ± 0,4	4,03 ± 0,3	3,60 ± 0,4	2,35 ± 0,4	2,89 ± 0,4	0–1** 0–2*** 0–3*** 0–4** 1–2** 1–3*** 2–3**
G-17 (μg/L)	6,60 ± 1,2	7,35 ± 0,7	5,71 ± 0,7	5,35 ± 0,7	NS	–	–	–	–	–	–

Significant differences *p < 0.05; **p < 0.01; ***p < 0.001; NS, Not significant.

**Table 5:** Endoscopy atrophy phenotypes (according to Kimura-Takemoto) in the study sub-groups.

Endoscopy atrophy phenotype (Kimura Takemoto)	Group 1 Atrophy IM-negative	Group 2 Atrophy IM-positive	Group 3 Atrophy coexisting dysplasia	*p*- value
Mild (C1-C2)	28 (54.9%)	19 (37.3%)	1 (2.5%)	1–3***, 2–3***
Moderate (C3-O1)	17 (33.3%)	29 (56.9%)	33 (82.5%)	1–3***, 2–3**
Severe (O2-O3)	6 (11.8%)	3 (5.9%)	6 (15.0%)	Not Significant

Significant differences *p < 0.05; **p < 0.01; ***p < 0.001.

**Table 6:** Endoscopy findings (according to Kimura-Takemoto) and gastritis OLGA and OLGIM stages.

Endoscopy atrophy phenotype (Kimura Takemoto)	OLGA staging				OLGIM staging				
	Stage I	Stage II	Stage III	Stage IV	Stage 0	Stage I	Stage II	Stage III	Stage IV
Mild (C1-C2)	19 (39.6%)	27 (56.3%)	2 (4.2%)	0 (0%)	28 (58,3**%**)	13 (27,1%)	7 (14,6%)	0	0
Moderate (C3-O1)	4 (5.1%)	24 (30.4%)	44 (55.7%)	7 (8.9%)	18 (22,8%)	1 (19,0%)	34 (43,0%)	9 (11,4%)	3 (3,8%)
Severe (O2-O3)	0 (0%)	3 (20.0%)	10 (66.7%)	2 (13.3%)	6 (40,0%)	2 (13,3%)	7 (46,7%)	0	0

The analysis of the correlation between the severity of gastric mucosal damage in CAG, determined endoscopically or histologically, and the results of the serological examination, found that simultaneously with the increase in the severity of CAG, the values of PG-I and PGI:PGII decrease and the values of serum NO and NO in gastric juice increase. The aforementioned correlation analysis found an inverse association, of medium intensity and statistically significant, between GCA forms, determined endoscopically according to the Kimura-Takemoto classification, and PG-I values (*ρ* = −0.46, *p* < 0.001), an inverse correlation, of medium intensity and statistically significant between CAG forms and PGI:PGII values (*ρ* = −0.48, *p* < 0.001), a direct correlation of medium intensity and statistically significant between CAG forms and serum NO values (*ρ* = 0.32, *p* < 0.001), a weak and statistically significant direct correlation between CAG forms and gastric juice NO values (*ρ* = 0.19, *p* < 0.05).

An association was revealed between the severity of atrophy according to the Kimura-Takemoto classification from C1 to O3 and the values of PG-I (inverse correlation, medium intensity and statistically significant, *ρ* = −0.34, *p* < 0.001), PGI:PGII (inverse correlation, of medium intensity and statistically significant, *ρ* = −0.33, p < 0.001), serum NO (direct correlation, of weak intensity and statistically significant, *ρ* = 0.23, *p* < 0.001) and NO in gastric juice (correlation direct, of weak intensity and statistically significant, *ρ* = 0.13, *p* < 0.05).

Serum level of PG-I (62.42 ± 2.5 μg/L and 51.33 ± 3.8 μg/L, respectively; *p* < 0.05) and PGI:PGII (4.61 ± 0.2 and 3, 75 ± 0.4, respectively; *p* < 0.01) represented statistically significantly higher data in patients with closed type GCA compared to patients with open type CAG, and the difference in serum PG-II level (14.41 ± 0.4 μg/L and 15.41 ± 1.1 μg/L, respectively; *p* > 0.05) between these two groups did not reach statistical certainty.

A significant inverse association emerged between the 3 phenotypical sub-groups of atrophic gastritis (sub-groups 1, 2, and 3 of the present study) and PG-I values (*ρ* = −0.52, *p* < 0.001), an inverse, statistically significant correlation between CAG forms and PGI:PGII values was determined (*ρ* = −0.64, *p* < 0.001), a direct, statistically significant correlation between forms of CAG and serum NO values (*ρ* = 0.85, *p* < 0.001), a direct, medium intensity and statistically significant correlation between forms CAG and NO values in gastric juice (*ρ* = 0.65, *p* < 0.001).

An association was revealed between the atrophy severity classified according to Kimura-Takemoto from C1 to O3, and PG-I values (inverse correlation, moderate intensity, and statistically significant, *ρ* = −0.34, *p* < 0.001), as well as PGI:PGII values (inverse correlation, moderate intensity, and statistically significant, *ρ* = −0.33, *p* < 0.001; Table 7).

**Table 7:** Serological examination results based on the forms of gastric mucosal atrophy, according to the Kimura-Takemoto endoscopic classification, in patients from study subgroup 1 (atrophy).

Serology	Endoscopy findings (according to Kimura-Takemoto)			
	Mild (C1-C2)	Moderate (C3-O1)	Severe (O2-O3)	*p*- value
PG-I (μg/L)	80,32 ± 3,5	59,29 ± 6,3	74,52 ± 13,9	1–2*
PG-II (μg/L)	13,40 ± 0,6	12,88 ± 1,5	20,55 ± 5,4	1–3*, 2–3*
PGI:PGII	6,32 ± 0,4	5,54 ± 0,8	4,10 ± 0,8	NS
G-17 (μg/L)	5,44 ± 0,8	7,51 ± 1,3	3,82 ± 1,3	NS

*p* < 0.05; **p < 0.01; ***p < 0.001.

## Discussion

Several studies have reported a consistent correlation between OLGA and OLGIM system stages and levels of PG-I and PGI:PGII. Serum mean PG-I and PGI:PGII values decrease significantly as OLGA and OLGIM stages increase (14, 19–25).

Another study from 2011, involving 269 patients with upper gastrointestinal symptoms and a mean age of 64.6 years, similarly observed a significant decrease in average PG-I values (from 82.1 μg/L in OLGA stage I to 64.3 μg/L in OLGA stage III-IV) and PGI:PGII (from 5.8 in OLGA stage I to 4.3 in OLGA stage III-IV). No correlations were found between PG-II and G-17 values with OLGA system stages (24).

In a 2017 study of 331 patients with upper gastrointestinal symptoms, with an average age of 53.1 years, researchers found a notable decrease in mean PG-I levels as the OLGA stages progressed. Specifically, PG-I levels dropped from 110.9 ± 47.5 μg/L in OLGA stage I to 66.6 ± 24.9 μg/L in OLGA stage IV. The PGI:PGII ratio also declined from 11.9 ± 3.7 in stage I to 5.7 ± 3.0 in stage IV. Our current study found similar trends using the OLGIM staging system, where mean PG-I levels decreased from 112.0 ± 51.2 μg/L in stage I to 92.8 ± 57.2 μg/L in stage IV, and the PGI:PGII ratio fell from 11.6 ± 4.9 to 6.7 ± 2.9 across the same stages (23).

The present study and analysis of serological parameters, based on OLGA system stages, showed a statistically significant decline in PG-I levels (from 79.48 ± 3.8 μg/L in stage I to 47.39 ± 10.1 μg/L in stage IV) and PGI:PGII values (from 6.58 ± 0.7 in stage I to 2.92 ± 0.2 in stage IV).

In the present study, we also performed a comparative analysis of OLGA stages and OLGIM stages. A similar study was recently performed by Lattore and al (26). One particularly that was found in our study group is that one patient was classified by the OLGA system in the low-risk category (stages I-II) and by the OLGIM system in the high-risk category (stages III-IV).

The frequency of diagnosing gastric atrophy based on serological parameters is influenced by the morphological type of gastric lesion. It decreases in patients with CAG without IM and GD but increases with the worsening morphological stages of CAG. A significant reduction in PG-I and PGI:PGII was observed alongside a significant increase in NO levels in blood serum and gastric juice with the advancement of OLGA and OLGIM stages. Serologically determined gastric atrophy is crucial for the non-invasive diagnosis and prognosis of CAG with IM and/or GD, both significant risk factors for gastric cancer development (23).

Although there is relatively good correlation among endoscopic, histological, and serological determinations of CAG, the sensitivity and specificity of these methods are not high, and histological diagnosis has its limitations. Hence, a multifactorial evaluation is necessary to enhance the accuracy of CAG diagnosis (23). For precise prediction of gastric cancer risk in clinical settings, CAG staging, including according to OLGA and OLGIM systems, should be combined with serum PG values (23).

In this study, we observed a notably high prevalence of dysplasia, with 40 out of 142 cases, which likely reflects an increased GC risk in the population studied. This elevated rate may be influenced by the regenerative changes associated with chronic HP infection. A further assessment by dysplasia grading was considered, but was deemed inappropriate due to the low number of patients with high-grade dysplasia (1).

The integration of the Kimura-Takemoto classification with GastroPanel biomarkers presents a promising approach to refining risk stratification for gastric cancer in patients with chronic atrophic gastritis. The Kimura-Takemoto system provides an endoscopic assessment of atrophic progression, distinguishing early (C1-C2) from advanced atrophy (C3-O3), while GastroPanel biomarkers—particularly low PG-I, a reduced PGI:PGII ratio, elevated G-17, and positive *Helicobacter pylori* serology—offer a complementary, non-invasive biochemical assessment of gastric mucosal health.

Patients classified as C3-O3 or demonstrating abnormal GastroPanel biomarker profiles should be considered high-risk for gastric cancer, aligning with OLGA/OLGIM stage III-IV classifications, which have been strongly associated with progression to intestinal-type gastric carcinoma. This raises a pertinent question regarding the necessity and extent of biopsy sampling in this cohort. Rather than performing extensive random biopsies, which may not significantly enhance diagnostic yield and could lead to unnecessary procedural burden, a targeted biopsy strategy may be more appropriate.

Specifically, biopsies should be directed toward regions with visible mucosal abnormalities, including areas of advanced atrophy, metaplasia, and dysplasia, as well as those showing irregularity in endoscopic texture and coloration. This targeted approach is consistent with the current paradigm shift in gastric cancer screening, which emphasizes precision and efficiency in surveillance. This approach was also described by other authors (27, 28).

Future studies should evaluate the effectiveness of this combined risk stratification model in guiding endoscopic biopsy protocols, optimizing diagnostic accuracy, and improving cost-effectiveness. If validated, this model could reduce unnecessary biopsies while ensuring early detection of high-risk lesions, ultimately contributing to better patient outcomes.

The study should be continued in the future in a longitudinal design, to assess the occurrence of gastric cancer in this cohort.

To our knowledge, this is the first application of OLGA and OLGIM staging in Moldovan patients. It validates the utility of these systems in Republic of Moldova.

## Conclusion

Gastric cancer is among the five most lethal epithelial malignancies. The OLGA and OLGIM staging systems categorize gastritis patients into five stages, each associated with significant differences in cancer progression. While both systems are reliable and effective for identifying individuals at higher risk of GC, their widespread use is still limited. Integrating the OLGA and OLGIM systems into real-world clinical practice could improve efforts in the secondary cancer prevention. This strategy may lead to a significant decrease in the incidence of invasive neoplastic disease and related mortality rates. This study applied gastritis staging to Moldovan gastritis patients. In this high-risk population, the study further supported the efficiency of gastritis staging in secondary GC prevention.

## Data Availability

The raw data supporting the conclusions of this article will be made available by the authors, without undue reservation.
